# Detection of Ganciclovir-Resistant Cytomegalovirus in a Prospective Cohort of Kidney Transplant Recipients Receiving Subtherapeutic Valganciclovir Prophylaxis

**DOI:** 10.1128/spectrum.02684-21

**Published:** 2022-06-06

**Authors:** Diana D. Wong, Wendy J. van Zuylen, Talia Novos, Sophie Stocker, Stephanie E. Reuter, Jane Au, Charles S. P. Foster, Richard O. Day, Andrea R. Horvath, Zoltan Endre, William D. Rawlinson

**Affiliations:** a Serology and Virology Division, NSW Health Pathology, Prince of Wales Hospital, Sydney, New South Wales, Australia; b School of Medical Sciences, Faculty of Medicine, University of New South Wales Sydney, Sydney, New South Wales, Australia; c Department of Chemical Pathology, New South Wales Health Pathology, Prince of Wales Hospital, Sydney, New South Wales, Australia; d Sydney Pharmacy School, Faculty of Medicine & Health, The University of Sydney, Sydney, New South Wales, Australia; e Department of Clinical Pharmacology & Toxicology, St Vincent’s Hospital, Sydney, New South Wales, Australia; f St Vincent’s Clinical School, Faculty of Medicine, The University of New South Wales, Sydney, New South Wales, Australia; g UniSA Clinical and Health Sciences, University of South Australia, Adelaide, South Australia, Australia; h Department of Nephrology, Prince of Wales Hospital, Sydney, New South Wales, Australia; i School of Biotechnology and Biomolecular Sciences, University of New South Wales Sydney, Sydney, New South Wales, Australia; Oklahoma State University, College of Veterinary Medicine

**Keywords:** ganciclovir, pharmacokinetics, cytomegalovirus, organ transplant, drug monitoring, resistance

## Abstract

Cytomegalovirus infection during antiviral prophylaxis occurs in transplant recipients despite individualized regimens based on renal function. Fifty kidney transplant recipients were assessed between 2016 and 2019 for valganciclovir dosing, ganciclovir exposure, cytomegalovirus infection, and genotypic resistance markers during the first year posttransplant. Ganciclovir plasma concentrations were measured using mass spectrometry. Population pharmacokinetics was used to determine individual ganciclovir exposure and to evaluate the ability of manufacturer dosing guidelines to meet therapeutic target daily area under the curve (AUC_24_) of 40 to 50 μg·h/mL. Full-length *UL54* and *UL97* were assessed using high-throughput sequencing in cytomegalovirus DNA-positive patient specimens. Valganciclovir doses administered to recipients with creatinine clearance of <40 mL/min were higher than specified by guidelines, and they were lower for recipients with creatinine clearance of ≥40 mL/min. The mean ganciclovir AUC_24_ was 33 ± 13 μg·h/mL, and 82% of subjects did not attain the therapeutic target. Pharmacokinetic simulations showed that the guidelines similarly could not attain the therapeutic target in 79% of individuals. Cytomegalovirus breakthrough occurred in 6% (3/50) of recipients, while 12% (6/50) developed late-onset infection. The mean AUC_24_s of recipients with (*n* = 3) and without (*n* = 47) infection were not significantly different (*P = *0.528). However, one recipient with an AUC_24_ of 20 μg·h/mL acquired two *UL97* ganciclovir resistance mutations. Current prophylaxis guidelines resulted in subtherapeutic ganciclovir exposure in several study recipients, including the emergence of resistance genotypes.

**IMPORTANCE** This study examined the pharmacokinetics and viral genomic data from a prospective cohort of kidney transplant recipients undergoing valganciclovir prophylaxis for cytomegalovirus (CMV) prevention. We showed for the first time using high-throughput sequencing the detection of ganciclovir resistance mutations in breakthrough CMV infection during subtherapeutic plasma ganciclovir as indicated by the pharmacokinetic parameter daily area under the curve (AUC_24_). In addition, we found that current valganciclovir dosing guidelines for CMV prophylaxis are predicted to attain therapeutic targets in only 21% of recipients, which is consistent with previous pharmacokinetic studies. The novel findings of resistance mutations during subtherapeutic ganciclovir exposure presented here can inform future studies investigating the dynamics of drug selection pressure and the emergence of resistance mutations *in vivo*.

## INTRODUCTION

The global seroprevalence of cytomegalovirus (CMV) in organ or blood donors is estimated at 86%, which is slightly higher than for the general population (1). In solid-organ transplantation, two-thirds of recipients develop CMV infection if not administered antiviral prophylaxis, predominantly as a result of immunosuppression and reactivation (2, 3). The clinical expression of CMV infection ranges from febrile illness to severe disease with high fever, organ failure, and death (4, 5). Use of valganciclovir, the oral prodrug of the guanosine analogue ganciclovir, is critical to prevent CMV infection in this setting (2).

Previous CMV breakthrough infections have been reported for a small subgroup of recipients across various transplant settings worldwide, despite the use of valganciclovir prophylaxis (6). This may be due to inadequate dosing for renal function or subtherapeutic drug exposure (7). Appropriate dosing is important for achieving efficacy while minimizing adverse effects such as neutropenia, thrombocytopenia, and the emergence of drug-resistant variants. Ganciclovir-resistant CMV isolates are difficult to treat without reducing immunosuppression and risking allograft loss, as current alternative drugs have serious adverse effects or are of limited availability (2, 8). Valganciclovir dosing for impaired renal function is provided by the manufacturer as a nomogram and described in the latest international consensus guidelines (2). Therapeutic drug monitoring for ganciclovir plasma concentration has been used to improve accuracy of drug dosing and, thus, improve clinical outcomes (9). A target plasma ganciclovir daily area under the curve (AUC_24_) of 40 μg·h/mL has been associated with a reduced risk of viremia in high-risk CMV-seronegative recipients who receive an organ from a CMV-seropositive donor (D+/R−) (10). While no toxicity target has been established, an upper limit of exposure of 50 μg·h/mL has been proposed (10). A previous trial reported that only 22% of patients achieved an AUC_24_ of 40 to 50 μg·h/mL with guideline-recommended dosing (11). This suggests that suboptimal drug exposure occurs in apparently appropriate valganciclovir treatment.

There are limited data to establish optimal valganciclovir prophylactic dosing in solid-organ transplant recipients (11, 12). It is unknown if kidney transplant recipients receiving valganciclovir prophylaxis exhibit therapeutic ganciclovir exposure. Furthermore, it is unknown if subtherapeutic exposure promotes the emergence of ganciclovir-resistant CMV variants, despite this being biologically plausible. The molecular determinants of ganciclovir resistance map to CMV genes *UL54* and *UL97* (8). Pharmacokinetic studies that analyze mutations at these genes fill a major gap in current knowledge. The purpose of this study was to evaluate valganciclovir prophylactic dosing and ganciclovir exposure in kidney transplant recipients and to assess concurrent CMV mutations.

## RESULTS

### Population characteristics and valganciclovir dosing.

**FIG 1:**
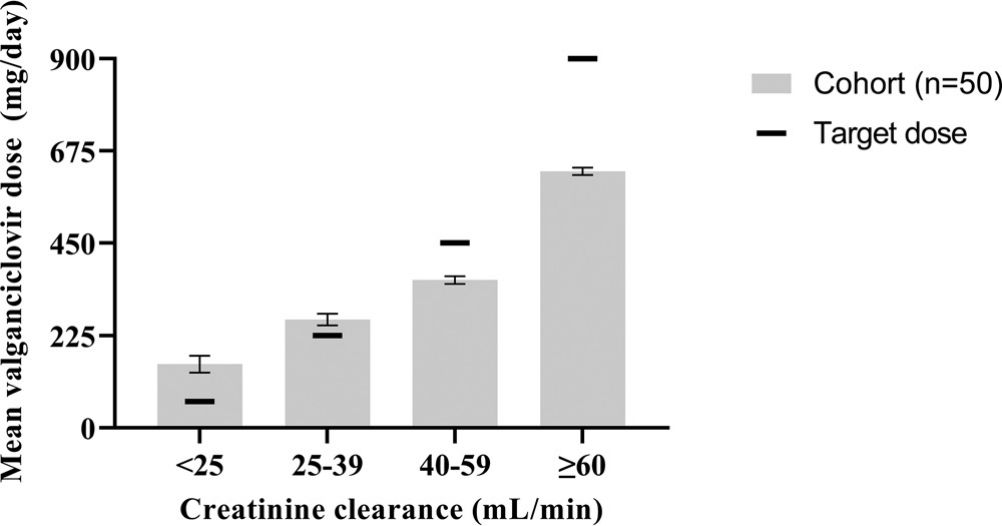
Valganciclovir dosing distribution according to creatinine clearance (7,501 data points shown). The black horizontal lines indicate the target dose as per hospital guidelines. The gray bars depict the means and 95% confidence intervals for daily valganciclovir dose received by the 50 recipients at different renal functions during the prophylaxis period.

The proportions of kidney transplant recipients (*n* = 50) who were D+/R−, D+/R+, and D−/R+ were 16%, 56%, and 26%, respectively (Table 1). The recipients’ average age was 52 years, and 40% were female (Table 1). Valganciclovir prophylaxis therapy was initiated on the first day after transplant in the vast majority. The duration of prophylaxis ranged from 2 to 12 months (Table 2). Three patients received valganciclovir for <3 months due to medical reasons with development of adverse effects (leukopenia), and none of these patients had a ganciclovir AUC_24_ above 50 μg·h/mL. The mean valganciclovir dose for CMV prophylaxis was 462 mg/day (range, 129 to 900 mg/day) (Table 2). Both “450 mg twice daily” and “900 mg once daily” were prescribed. This did not affect the predicted AUC_24_ since daily drug exposure would be the same for both regimens. The dosing distribution of valganciclovir across recipients stratified by renal function is summarized in Fig. 1. Doses of valganciclovir administered were greater than guidelines during moderate (creatinine clearance [CL_CR_], 25 to 39 mL/min) and severe (CL_CR_, <25 mL/min) renal function impairment. In contrast, doses lower than recommended were administered during mild renal impairment (CL_CR_, 40 to 59 mL/min) and normal renal function (CL_CR_, ≥60 mL/min) (Fig. 1).

**TABLE 1:** Demographic characteristics of study subjects

Characteristic	Value for transplant recipients (*n* = 50)
Sex, no. (%)	
Female	20 (40)
Male	30 (60)
	
Age, yrs	
Mean	52
SD	13
	
Donor, no. (%)	
Living related/unrelated	9 (18)/9 (18)
Deceased donation after brain death/donation after cardiac death	22 (44)/9 (18)
Unknown	1 (2)
	
HLA-A mismatches, no. (%)	
No mismatch	9 (18)^*a*^
1 or 2 mismatches	40 (82)^*a*^
	
HLA-B mismatches, no. (%)	
No mismatch	5 (10)^*a*^
1 or 2 mismatches	44 (90)^*a*^
	
HLA-DR mismatches, no. (%)	
No mismatch	10 (20)^*a*^
1 or 2 mismatches	39 (80)^*a*^
	
Donor-specific antibody, no. (%)	
Present	23 (47)^*a*^
Class I	10 (20)^*a*^
Class II	10 (20)^*a*^
Classes I and II	3 (6)^*a*^
Nil	26 (53)^*a*^
	
Donor/recipient CMV IgG at transplant, no. (%)	
D+/R−	8 (16)^*a*^
D+/R+	28 (56)^*a*^
D−/R+	13 (26)^*a*^
D unknown/R+	1 (2)
	
Induction immunosuppression, no. (%)	
Anti-thymocyte globulin	6 (12)
Basiliximab	43 (86)
Missing	1 (2)
	
Maintenance immunosuppression, no. (%)	
Mycophenolate + prednisolone + calcineurin inhibitors	50 (100)

a *n* = 49 due to missing data for one recipient whose transplant was performed overseas.

**TABLE 2:** Valganciclovir dosing history and Bayesian estimation of average ganciclovir AUC_24_s of kidney transplant recipients (*n* = 50)

Parameter^*a*^	Duration of prophylaxis (mo)	Total valganciclovir (g)	Avg valganciclovir (mg/day)	AUC_24_ (μg·h/mL)^*b*^
Mean	5	71	462	33
SD	3	62	250	13
Min	2	11	129	11
Median	4	47	435	30
Max	12	322	900	62

a Min, minimum; Max, maximum.

b Average ganciclovir exposure over the course of prophylaxis therapy or until CMV viremia was first detected.

### Ganciclovir AUC_24_ in study cohort and dosing simulation.

A total of 224 blood samples were obtained from recipients receiving valganciclovir prophylaxis. Sampling of blood at 0, 1, and 2 h postdose was successfully achieved for 45 recipients. Four participants who had at least one blood sample missing and one recipient who did not have any blood samples available were included in the study. A full listing of each blood collection for recipients is shown in Table S2 in the supplemental material.

The predicted mean ganciclovir AUC_24_ during prophylaxis was 33 ± 13 μg·h/mL (range, 11 to 62 μg·h/mL) (Table 2). The ganciclovir AUC_24_s for male (*n* = 30) and female (*n* = 20) recipients were 31 ± 14 μg·h/mL and 37 ± 12 μg·h/mL, respectively (Student’s *t* test, *P = *0.132). There was no correlation observed between ganciclovir trough and AUC_24_ values among study recipients (*R*^2^ = 0.0001; *P = *0.94), and data are not presented.

Given that valganciclovir dosing was discordant with guidelines, pharmacokinetic simulations were conducted via readjustment of all patient doses after each dosing interval to determine if the manufacturer’s recommended dosing would be predicted to result in higher therapeutic target attainment than that seen in the study population. Simulations indicated that if recipients had been dosed with valganciclovir using the manufacturer’s nomogram, there would have been minimal change in the proportion of recipients with ganciclovir exposure within 40 to 50 μg·h/mL (Table 3). However, the proportion of recipients with an AUC_24_ of <40 μg·h/mL was reduced (chi-squared test, *P = *0.005) and the proportion of recipients with an AUC_24_ of >50 μg·h/mL was increased (chi-squared test, *P = *0.005) (Table 3).

**TABLE 3:** Proportion of individuals achieving ganciclovir therapeutic AUC_24_ values between the study cohort and simulation to match the manufacturer’s dosing guidelines

Regimen	% of subjects achieving therapeutic AUC_24_		
	<40 μg·h/mL	40–50 μg·h/mL	>50 μg·h/mL
Study cohort (*n* = 50)	68	18	14
Simulation (*n* = 50,000)	42	21	37

### CMV infection during prophylaxis.

There were 3/50 recipients with breakthrough CMV infection, which occurred while they were on prophylaxis. One of these three developed clinically diagnosed CMV disease (Table 4).

**TABLE 4:** Detectable CMV viremia or disease and the associated *UL97* and *UL54* single nucleotide polymorphisms of recipients in this study^*a*^

CMV IgG	Days^*b*^	Viremia, IU/mL^*c*^	Disease, IU/mL^*c*^	Symptoms	SNP(s) in:	
					*UL97*	*UL54*
During prophylaxis						
D+/R−	63	3,340		Asymptomatic	Q19E, T75A, S108N, Q126L, **A594V**, **C603W**	A647V, N898D, A1108T, T1122A
D+/R+	75	<250		Asymptomatic	—	—
D+/R−	114		Esophagitis (<250)	Odynophagia, esophageal ulceration, intermittent fever	—	—
Postprophylaxis during the first yr of transplant						
D+/R−	250		Colitis (33,050)	Diarrhea, vomiting	T75A	S655L, N685S, L897S, N898D, R984H, A1108T
D+/R−	208		Gastritis (2,720)	Nausea, reduced oral intake	T75A	N898D, A1108T, S1235T
D+/R−	358		Syndrome (236,000)	Systemic symptoms of leukopenia, transaminitis, diarrhea	T75A, Q126L, ***H469Y***	A614V, A1108T
D+/R−	293		Colitis (312,500)	Vomiting, diarrhea, fever	T75A, R112C, R112H, Q126L	S24L, S655L, N685S, G874R, L897S, N898D, A1108T, T1122A
D+/R+	205	<250		Asymptomatic	—	—
D+/R+	175	<250		Asymptomatic	—	—

a *UL97* and *UL54* reads were mapped against the CMV strain Merlin genome to assign variants (GenBank accession number NC_006273). Ganciclovir resistance mutations are shown in bold, mutations associated with ganciclovir resistance are shown in bold with italics, and mutations with unknown resistance phenotypes are underlined. —, gene not amplified or detected upon sequencing data analysis.

b Days posttransplant when viremia was first detected.

c Peak viral load is shown.

**FIG 2:**
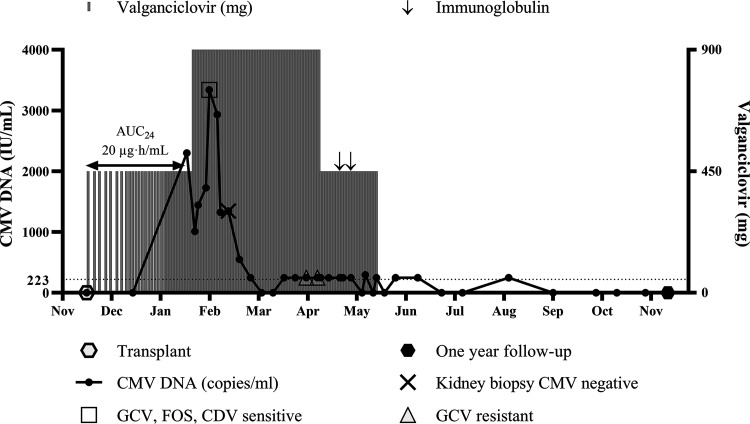
Clinical course of kidney transplant recipient who developed CMV infection during prophylaxis and subsequent ganciclovir resistance. The CMV assay detection limit was 223 IU/mL (dotted line). GCV, ganciclovir; FOS, foscarnet; CDV, cidofovir.

The first recipient developed asymptomatic primary CMV at day 63 posttransplant (Table 4). Valganciclovir was increased from 450 mg to 900 mg daily on day 67 posttransplant, with a reduction in prednisone (Fig. 2). Drug resistance testing using Sanger sequencing on day 77 posttransplant returned no detectable resistance mutations for ganciclovir, foscarnet, or cidofovir. A decrease in CMV viral load was observed, although the titer was detectable at low counts (Fig. 2). A kidney biopsy specimen taken on day 89 posttransplant did not show CMV on histological staining and immunohistochemistry. Prophylaxis was stopped at 6 months posttransplant. Over this time, the patient had received two rounds of immunoglobulin (Privigen) transfusions (Fig. 2). The ganciclovir AUC_24_ for this subject was 20 μg·h/mL.

The second recipient exhibited <250 IU/mL of CMV DNA once on day 75 posttransplant and was asymptomatic (Table 4). No changes to valganciclovir or immunosuppression dosages were made. The ganciclovir AUC_24_ for this subject was 22 μg·h/mL.

The third recipient was diagnosed with CMV esophagitis disease (Table 4). Biopsies of the ulcer and esophageal tissue returned negative for CMV immunohistochemistry and CMV PCR. CMV esophagitis remained the differential diagnosis, which was confirmed by the appearance on endoscopy. Cytomegalovirus DNA at <250 IU/mL was detected in the plasma on day 114 posttransplant (Table 4). Testing for other infectious agents, including serum herpes simplex virus, varicella-zoster virus, Mycobacterium tuberculosis, and adenovirus, returned negative. The patient responded slowly to valganciclovir and intravenous ganciclovir for 4 weeks, with reduction in mycophenolate and tacrolimus, before switching to valganciclovir prophylaxis for 6 months. The ganciclovir AUC_24_ for this subject was 42 μg·h/mL.

### CMV infection postprophylaxis.

Within the first year of transplantation, 6/50 (12%) recipients developed CMV infection after discontinuing prophylaxis; 4/6 of these recipients developed CMV disease and required therapy (Table 4). All recipients responded to therapy, which typically consisted of intravenous ganciclovir or valganciclovir. Two recipients experienced asymptomatic low-grade viremia in which no clinically diagnosed disease related to CMV occurred (Table 4). None of these patients experienced breakthrough CMV during valganciclovir prophylaxis.

### Detection of SNP from amplicon-based high-throughput sequencing.

A total of 49 clinical specimens that were positive for CMV by quantitative PCR or histology were collected during the follow-up period. The PCRs for *UL54* and *UL97* showed detectable amplicons in 20/49 and 44/49 samples, respectively. Representative gels are presented in Fig. S1. The MiSeq sequencing run generated 1,740,262 reads in total, with a mean of 23,839 ± 4,560 reads per sample. Mapping of the *UL54* and *UL97*-generated reads to the Merlin reference genome showed successful alignment for 20/20 and 24/44 of the amplicons, respectively. Samples which did not align successfully to the reference genome were excluded from the single-nucleotide polymorphism (SNP) analysis. There were 3/50 recipients who harbored mutations associated with ganciclovir resistance or an unknown drug resistance phenotype (Table 4). The ganciclovir resistance mutations *UL97* A594V and *UL97* C603W were detected in one recipient sampled on days 138 and 145 posttransplant, respectively (Fig. 2). Those samples were collected from this recipient during valganciclovir treatment for CMV breakthrough during prophylaxis where the ganciclovir AUC_24_ was 20 μg·h/mL, with viral loads of <250 IU/mL. The mutation *UL97* H469Y, which is linked to but is not known to directly confer ganciclovir resistance, and the mutation *UL54* A614V, of unknown drug resistance phenotype, were detected in a second recipient with CMV syndrome at 1 year posttransplant (Table 4). The mutation *UL54* R984H, of unknown resistance phenotype, was detected in a third recipient diagnosed with CMV colitis at 250 days posttransplant (Table 4). Full variant profiles containing polymorphisms in all samples tested are summarized in Table S3.

## DISCUSSION

This is the first and largest prospective study to provide ganciclovir measurement in blood via AUC_24_ that was undertaken alongside evaluation of the emergence of CMV antiviral resistance to assess the impact of dosing variation post-renal transplantation on CMV gene mutation. Our finding of discordant dosing to guidelines in clinical practice may have resulted from clinical therapeutic decisions, dose interval prolongation, inaccurate estimates of glomerular filtration rate (GFR), and changes in renal clearance over time. Therapeutic decisions outside the dosing guidelines are known to occur (2, 7). Methods for estimating GFR to adjust doses, such as the Modification of Diet in Renal Disease (MDRD) and Chronic Kidney Disease Epidemiology Collaboration (CKD-EPI) equations, have been shown elsewhere (13, 14). Despite CKD-EPI being superior in the clinical setting, the Cockcroft and Gault (C-G) formula remains the current recommended formula for estimating renal function for valganciclovir dosing and was adhered to in this study.

The mean AUC_24_ of our study was lower than found in previous studies, which is likely due to administration of lower-than-recommended valganciclovir doses (6). In the simulation experiment, predictions indicated that 100%-matched guideline dosing would still not have achieved the therapeutic target in 79% of study individuals. This may, at least in part, be due to substantial temporal changes in renal function seen in recipients throughout the course of therapy posttransplant and to the inability of dose adjustment strategies to adequately account for this changing renal function (7). Our findings are comparable with those of previous studies, with one study recommending higher doses of valganciclovir corrected to renal function to achieve therapeutic targets (11, 12). However, this type of frequency of renal function monitoring and dose adjustment would be unlikely in a clinical scenario, and emphasis on the importance of follow-up is appropriate.

In this study, the incidence of CMV breakthrough, 6%, was higher than for most cohorts, for which rates were reported between 0% and 6.1% (6). One study reported an exceptionally high incidence, 50%, due to significant underdosing resulting from inaccurate estimation of the GFR, highlighting the importance of dosing that takes into account an accurate measure of glomerular filtration (14). Ganciclovir is activated by the CMV *UL97*-encoded protein kinase to target viral chain extension at the CMV *UL54*-encoded polymerase complex. The sole recipient who acquired ganciclovir resistance mutations at CMV *UL97* in this study had the lowest AUC_24_, 20 μg·h/mL, among D+/R− recipients. The canonical ganciclovir resistance mutations *UL97* A594V and C603W (which also confer low-grade cross-resistance to maribavir and cyclopropavir) were detected in low-viral-copy-number specimens due to the sensitivity of the sequencing platform used in this study (8). A previous multicenter study that analyzed 239 D+/R− recipients reported an absence of resistance mutations during valganciclovir prophylaxis while sequencing *UL54* and *UL97* using the Sanger method (15). Future work to expand to whole-genome sequencing of the ~240-kb CMV genome may uncover resistance mutations beyond the current examined genes (8, 16).

The incidence of late-onset CMV disease in our study was 8%, comparable to that found in systematic literature reviews, reported at 8.9% (3). All four cases were D+/R− and the recipient with the lowest AUC_24_ was the first to develop CMV disease. The potential for ganciclovir exposure to influence late-onset CMV disease remains uncertain.

There are some limitations to this study. There was a higher representation of males and lower proportion of D+/R− recipients in an otherwise homogenous cohort. The sample size could not establish a causal relationship between AUC_24_ and efficacy. Obtaining three blood samples in a single dosing interval was feasible for most recipients. However, participation rates were reduced after the first month of transplant due to recipients returning to local hospitals after discharge from our center. Blood samples were collected at specified times (e.g., week 1) for some patients, and the prophylactic duration varied between patients. However, population pharmacokinetic modeling took these factors into account to allow determination of drug exposure over the entire prophylactic course, enabling direct comparisons in this study. Future studies should investigate how to reduce blood sampling further without compromising the accuracy of estimates of drug exposure (17).

In conclusion, this study highlights evidence of subtherapeutic ganciclovir exposure and the clinical consequences of CMV breakthrough and acquisition of resistance. There is potential utility for ganciclovir therapeutic drug monitoring and determination of AUC_24_ to identify those patients at greater risk, for whom earlier testing for viral breakthrough and resistance variants could be deployed.

## MATERIALS AND METHODS

### Study population.

This single-center, prospective, observational study was conducted at Prince of Wales Hospital (POWH), Australia, from July 2016 to October 2019. The study was approved by the NSW Government Health South Eastern Sydney Local Health District Human Research Ethics Committee (LNR/16/POWH/307). All participants provided written informed consent. Adults (≥18 years) who had undergone kidney transplantation and required valganciclovir prophylaxis were eligible to participate. Patients were excluded if participation was considered unsuitable (due to medical concerns) by the attending physician. Additional data were collected from electronic medical records: gender, weight, age, serum creatinine, CMV data (viral load, IgG, biopsy, and clinical symptoms), concomitant immunosuppressive medications, and donor transplant data (living or deceased, CMV IgG, HLA mismatches, and donor-specific antibody). Patients received valganciclovir doses that were decided by the attending physician. Doses were adjusted to renal function using the Cockcroft and Gault (C-G) formula according to POWH renal transplant guidelines that mirrored those in the manufacturer’s product information (Table S1). All valganciclovir doses were recorded for each subject.

### Specimen processing and ganciclovir assay.

A limited blood sampling strategy at predose and 1 h and 2 h postdose was used to determine AUC_24_ (18). Blood samples were obtained at steady state after at least 3 days of prophylaxis treatment. Steady state was considered achieved after 5 half-lives (determined for each recipient) at the same dose. Sampling occasions occurred at weeks 1, 2, and 3 to 4, month 2, and month 3 posttransplant. Patients were provided with the option to participate in any one or all of the proposed blood collection occasions. Given the lack of intraindividual variability in pharmacokinetics for ganciclovir, the approach undertaken was appropriate to address the study objective and consistent with that undertaken for the evaluation of drug exposure in other therapeutic domains (19).

Blood EDTA samples were centrifuged at 600 × *g* for 15 min, and plasma aliquots were stored at −80°C. Ganciclovir concentrations were measured using liquid chromatography-tandem mass spectrometry as described by Heinig et al. (20). Chromatographic separation was performed using a Luna 3-μm Silica (2) 50- by 2.0-mm analytical column (Phenomenex, Australia). Ganciclovir and ganciclovir-d5 were monitored at ion transitions 256.19→134.90 *m/z* and 261.19→152.00 *m/z*, respectively. All samples were run in singlet. Assay validation was in accordance with the National Pathology Accreditation Advisory Council (21). Linearity was between 0.05 and 20 μg/mL, with intra-assay and interassay precision of <15%. The lower limit of quantification was 0.05 μg/mL.

### AUC_24_ determination and regimen evaluation.

Pharmacokinetic parameters were calculated using empirical Bayesian estimation based on a two-compartment population pharmacokinetic model (22). Analysis included determination of average ganciclovir AUC_24_ over the prophylactic course for each individual.

A population pharmacokinetic approach was used to assess the ability of the manufacturer’s dosing guidelines to achieve target ganciclovir exposure. Monte Carlo simulations were used to provide predictions of average AUC_24_ over the treatment course for 1,000 *in silico* data sets based on the study population, from which the proportion of individuals predicted to attain an average AUC_24_ within a target range of 40 to 50 μg·h/mL was determined.

### CMV detection and amplification of *UL54* and *UL97*.

Recipients were tested for CMV at the discretion of their physician. The diagnostic assay included CMV R-GENE (bioMérieux, Australia) and the LightCycler 480 real-time PCR 2.0 system (Roche, Australia). The cutoff value for positivity provided by the manufacturer was 223 IU/mL. CMV-positive samples were stored at −80°C. After follow-up, nucleic acids were extracted with the MagNAPure96 DNA and ViralNA small-volume kit on a MagNAPure96 instrument (Roche). Full-length CMV *UL54* and *UL97* were amplified using nested PCR with Platinum SuperFiIIGreen master mix (Invitrogen, Australia) (23, 24). The primers used for gene amplification were UL97 EX 1, UL97 EX 2, UL97 INT 1, and UL97 INT 2 for *UL97* and UL54-Ext.1, UL54-Ext.2, UL54-Int.1, and UL54-Int.2 for *UL54*; their sequences are published elsewhere (23, 24). Gel electrophoresis was used to screen amplicons of correct size (23, 24). Amplicons were purified by sample purification beads (Illumina, Australia) at a 1.8× (vol/vol) ratio.

### Library preparation for sequencing on Illumina platforms.

Purified DNA was quantified using the Quant-iT PicoGreen double-stranded DNA (dsDNA) kit (Invitrogen, Australia), with fluorescence measured on a Victor X2 plate reader (PerkinElmer, Australia). Dual-indexed sequencing libraries were prepared using DNA Prep v2 (Illumina, Australia). The size distribution of the library was assessed on LabChip GX Touch24, using the HTDNA high-sensitivity assay (PerkinElmer). Samples were pooled in equimolar quantities and spiked with 10% PhiX. Sequencing was performed using MiSeq reagent Nano v2 on a MiSeq sequencer (Illumina).

### Data analysis.

Residual sequencing adapters were removed from raw reads before filtering for quality and length using *fastp* (25). Reads were aligned to the Merlin reference genome and sequence data predominately comprising nonspecific amplification of human DNA were identified using karaken 2 and excluded from analysis (26). Clean reads were mapped against the CMV Merlin genome (GenBank accession number NC_006273.2) using *bwa-mem* v0.7.17-r1188 (27, 28). Amplicon primers were soft-clipped from the alignment using *iVar* (29). Variants were called using *samtools mpileup* v1.10 (30) and *iVar* v1.3 (29), with minimum thresholds for PHRED quality, read depth, and allele frequency set at 20, 10, and 0.1, respectively. Variants were annotated using snpEff 5.0c (31). Nonsynonymous amino acid replacements were detected by parsing the output with a custom python3 script.

Population pharmacokinetic modeling and simulation were conducted using NONMEM VIII (ICON Development Solutions, USA) with an Intel Fortran compiler (Intel Visual Fortran Composer XE 2013) and Wings for NONMEM7 interface (http://wfn.sourceforge.net). Data processing was conducted using R version 3.3.2 (R Foundation for Statistical Computing).

### Statistical analysis.

The proportions of individuals at AUC_24_ targets were summarized by counts and descriptive statistics. Comparisons were made between regimens using Pearson’s chi-squared test. An independent two-tailed *t* test was used to compare mean AUC_24_s. Linear regression was used to examine correlation between trough and AUC_24_.

### Data availability.

The raw sequencing reads are available from the Sequence Reads Archive (SRA) under accession number PRJNA791463.
