# Supplementary material for: Detection of Ganciclovir-Resistant Cytomegalovirus in a Prospective Cohort of Kidney Transplant Recipients Receiving Subtherapeutic Valganciclovir Prophylaxis
Source: Microbiol Spectr. 2022 Jun 6;10(3):e02684-21. doi: 10.1128/spectrum.02684-21 (PMC9241781; doi:10.1128/spectrum.02684-21)
Supplement: Supplemental file 1 — Tables S1 to S3; Fig. S1. Download spectrum.02684-21-s0001.pdf, PDF file, 0.5 MB [file spectrum.02684-21-s0001.pdf]

Supplementary Table 1. Valganciclovir (tablet formulation) prophylaxis dosing guidelines for recipients with impaired renal function.

Creatinine clearance (mL/min)	Prince of Wales[a] (mg)	Manufacturer (mg)
≥60	900 daily	900 daily
40 – 59	450 daily	450 daily
25 – 39	450 every 2 days	450 every 2 days
10 – 24	450 twice weekly	450 twice weekly
<10	450 twice weekly	Not recommended

*Calculated from serum creatinine by the Cockcroft-Gault formula.

[a]Recommended durations of therapy for D+/R- and R+ recipients were six months and three months, respectively.

Supplementary Table 2. Blood collection used for determining ganciclovir concentration for participants in this study.

Subject	Days post-transplant	Trough	1-hour	2-hour
1	13	×	×	×
	30	×	×	×
	60	×	×	×
	88	×		
2	5	×	×	×
	13	×	×	×
3	16	×	×	×
4	3	×	×	×
	11	×	×	×
	72	×	×	×
5	11			×
	18	×	×	×
6	6	×	×	×
7	9	×	×	×
	17	×	×	×
	66	×		
8	4	×	×	×
	15	×	×	×
9	5	×	×	×
10	4	×	×	×
	14	×	×	×
11	5	×	×	×
12	6	×	×	×
13	4	×	×	×
14	5	×	×	×
15	66	×	×	×
16	7	×	×	×
17	14	×	×	×
18	25			
19	21	×	×	×
20	6	×	×	×
	13	×	×	×
21	5	×	×	×
	14			×
22	8	×	×	×
23	5	×		×
24	8	×		
	9	×	×	×
	27	×	×	×
	62			×
	77	×		
25	8	×	×	×
26	5	×	×	×

	12	×	×	×
27	8	×	×	×
	14	×	×	×
	28	×	×	×
	59	×	×	×
	89	×	×	×
28	7	×	×	×
	15	×	×	×
29	9	×	×	×
30	6	×	×	
31	17	×	×	×
32	5	×		
	8		×	×
	15		×	
	31	×	×	×
	61	×	×	×
	90	×	×	×
33	7	×	×	×
	13			
	30	×	×	×
	58	×	×	×
	89	×	×	×
34	5	×	×	×
35	7	×	×	×
	14	×	×	×
36	5	×		
37	39	×	×	×
38	4	×	×	×
39	4	×	×	×
40	4		×	×
	8	×		
41	3	×	×	×
42	4	×	×	×
43	4	×		×
44	4	×	×	×
45	4	×	×	×
	7			×
	14		×	
	30			×
	63			×
	85			×
46	3	×	×	×
47	4	×	×	×
48	5	×	×	×
49	7	×	×	×
50	5	×	×	×

Supplementary Table 3. Genomic profile of major CMV variants from transplant recipients in this study.

Sample No.	Subject	Days post-transplant	Specimen type	CMV (DNA IU/mL)	UL97 PCR	Sequence aligned[a]	UL97 variants	UL54 PCR	Sequence aligned[a]	UL54 variants
1	Late onset CMV #1	250	Plasma	20750	+	+	T75A	+	+	S655L, N685S, L897S, N898D, R984H, A1108T
2		261	Plasma	31550	+	+		+	+	S655L, N685S, L897S, N898D, A1108T
3		264	Plasma	7500[b]	+	+	T75A	+	+	S655L, N685S, L897S, N898D, A1108T
4		264	Tissue	CMV colitis	+	+	T75A	+	+	S655L, N685S, L897S, N898D, A1108T
5		264	Tissue	CMV colitis	+	+	T75A	+	+	S655L, N685S, L897S, N898D, A1108T
6		264	Tissue	Normal mucosa	+	+	T75A	+	+	S655L, N685S, L897S, N898D, A1108T
7		264	Tissue	Normal mucosa	+	+	T75A	+	+	S655L, N685S, L897S, N898D, A1108T
8		265	Plasma	33050	+	+	T75A	+	+	S655L, N685S, L897S, N898D, A1108T
9		268	Plasma	<250	+	-	N/A	-	N/A	N/A
10	Late onset CMV #5	205	Plasma	<250	+	-	N/A	-	N/A	N/A
11	Late onset CMV #2	208	Plasma	2720	+	+		+	+	N898D, A1108T, S1235T
12		211	Tissue	CMV gastritis	+	+	T75A	+	+	N898D, A1108T, S1235T
13		229	Plasma	<250	+	-	N/A	-	N/A	N/A
14	Late onset CMV #6	175	Plasma	<250	+	-	N/A	-	N/A	N/A
15	CMV during prophylaxis #1	63	Plasma	2300	+	+	Q19E, T75A, S108N, Q126L	+	+	A647V, N898D, A1108T, T1122A
16		68	Plasma	1010	+	+	Q19E, T75A, S108N, Q126L	+	+	A647V, N898D, A1108T, T1122A
17		70	Plasma	1445	+	-	N/A	+	+	A647V, N898D, A1108T, T1122A

18		75	Plasma	1730	+	+	Q19E, T75A, S108N, Q126L	-	N/A	N/A
19		77	Plasma	3340[b]	+	+	Q19E, T75A, S108N, Q126L	-	N/A	N/A
20		82	Plasma	2935	+	+	Q19E, T75A, S108N, Q126L	+	+	A647V, N898D, A1108T, T1122A
21		84	Plasma	1325	+	+	Q19E, T75A, S108N, Q126L	-	N/A	N/A
22		89	Plasma	1345	+	+	Q19E, T75A, S108N, Q126L	-	N/A	N/A
23		96	Plasma	550	+	-	N/A	-	N/A	N/A
24		103	Plasma	<250	+	-	N/A	-	N/A	N/A
25		124	Plasma	<250	+	-	N/A	-	N/A	N/A
26		131	Plasma	<250	+	-	N/A	-	N/A	N/A
27		138	Plasma	<250	+	+	Q19E, T75A, S108N, Q126L, **A594V**	-	N/A	N/A
28		145	Plasma	<250	+	+	Q19E, T75A, S108N, Q126L, **C603W**	-	N/A	N/A
29		147	Plasma	<250	+	-	N/A	-	N/A	N/A
30		152	Plasma	<250	+	-	N/A	-	N/A	N/A
31		159	Plasma	<250	-	-	N/A	-	N/A	N/A
32		161	Plasma	<250	+	-	N/A	-	N/A	N/A
33		166	Plasma	<250	-	-	N/A	-	N/A	N/A
34		175	Plasma	295	+	-	N/A	-	N/A	N/A
35		182	Plasma	<250	+	-	N/A	-	N/A	N/A
36		194	Plasma	<250	-	-	N/A	-	N/A	N/A
37		208	Plasma	<250	+	-	N/A	-	N/A	N/A
38		265	Plasma	<250	-	-	N/A	-	N/A	N/A
39	CMV during prophylaxis #2	75	Plasma	<250	+	-	N/A	-	N/A	N/A
40	CMV during prophylaxis #3	114	Plasma	<250	+	-	N/A	-	N/A	N/A
41	Late onset CMV #3	360	Plasma	236000	+	+	T75A, Q126L, ***H469Y***	+	+	A614V, A1108T
42		364	Plasma	12150	+	+	T75A, Q126L, ***H469Y***	+	+	A614V, A1108T

43		368	Plasma	25950	+	+	T75A, Q126L, ***H469Y***	+	+	A614V, A1108T
44		375	Plasma	920	+	-	N/A	-	N/A	N/A
45	Late onset CMV #4	293	Plasma	169000	+	+	T75A, R112C, R112H, Q126L	+	+	S24L, S655L, N685S, G874R, L897S, N898D, A1108T, T1122A
N/A		298	Plasma[c]	312500[b]	N/A	N/A	N/A	N/A	N/A	N/A
46		305	Plasma	21600	+	+	T75A, R112C, R112H, Q126L	+	+	S24L, S655L, N685S, G874R, L897S, N898D, A1108T, T1122A
47		312	Plasma	3050	+	-	N/A	+	+	S24L, S655L, N685S, G874R, L897S, N898D, A1108T, T1122A
48		321	Plasma	<250	+	-	N/A	-	N/A	N/A
49		295	Tissue	CMV colitis	-	-	N/A	-	N/A	N/A

[a]Sequencing reads generated from the positive PCR amplicons were aligned to the Merlin reference genome. Samples were designated as sequencing positive if >1% of sequencing reads aligned to the reference genome. Only reads that aligned to the Merlin reference genome were included when analysing CMV-sequence positive samples.

[b]Sanger sequencing antiviral resistance test showed susceptible to ganciclovir, foscarnet, and cidofovir.

[c]Sample missing.

UL97 and UL54 reads were mapped against the CMV strain Merlin genome to assign variants (NCBI Accession number NC_006273) [23]. Unknown resistance phenotype shown underlined; ganciclovir-resistance shown in bold; associated with ganciclovir-resistance shown in bold with italics; N/A, not applicable.

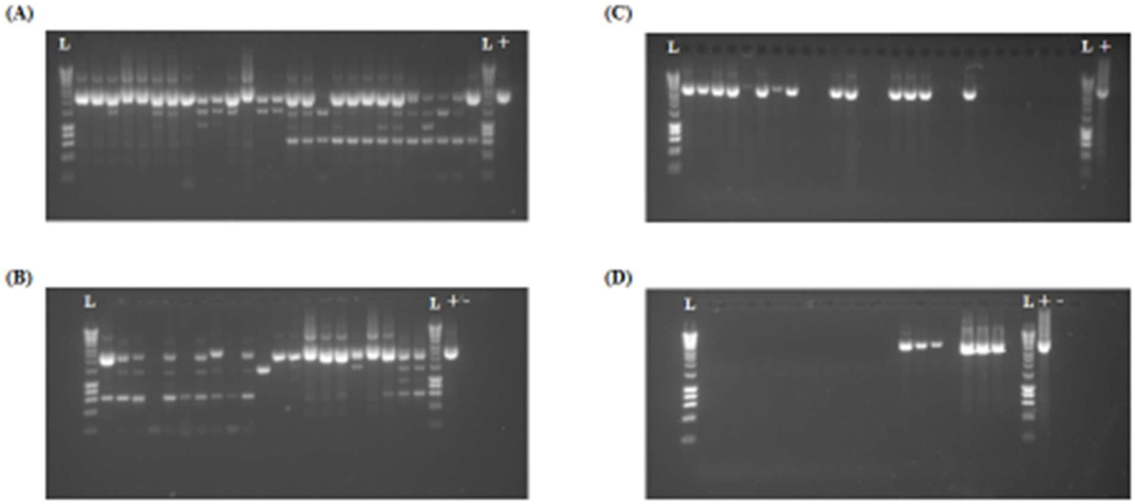

Supplementary Figure 1. Gel electrophoresis pictures of second round amplicons of nested PCR of CMV positive clinical specimens (A) UL97 samples #1-27, (B) UL97 samples #28-48, (C) UL54 samples #1-27, (D) UL54 samples #28-48. A 1 kb ladder (L) was used (Hyperladder, Bioline) was used. Positive control (+), CMV (Merlin strain). Negative control (-), water.
